# Effects of Texture Component Orientation on Orientation Flow Visibility for 3-D Shape Perception

**DOI:** 10.1371/journal.pone.0053556

**Published:** 2013-01-03

**Authors:** Michelle L. Fowler, Andrea Li

**Affiliations:** 1 Neuropsychology Doctoral Subprogram, City University of New York Graduate Center, New York, New York, United States of America; 2 Department of Psychology, Queens College, Flushing, New York, United States of America; University College London, United Kingdom

## Abstract

In images of textured 3-D surfaces, orientation flows created by the texture components parallel to the surface slant play a critical role in conveying the surface slant and shape. This study examines the visibility of these orientation flows in complex patterns. Specifically, we examine the effect of orientation of neighboring texture components on orientation flow visibility. Complex plaids consisting of gratings equally spaced in orientation were mapped onto planar and curved surfaces. The visibility of the component that creates the orientation flows was quantified by measuring its contrast threshold (CT) while varying the combination of neighboring components present in the pattern. CTs were consistently lowest only when components closest in orientation to that of the orientation flows were subtracted from the pattern. This finding suggests that a previously reported frequency-selective cross-orientation suppression mechanism involved with the perception of 3-D shape from texture is affected by proximity in orientation of concurrent texture components.

## Introduction

**Figure 1:**
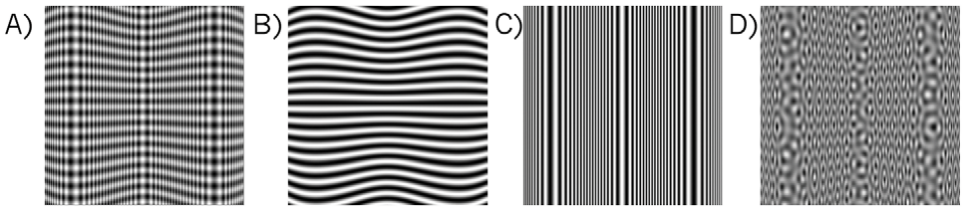
Orientation flows and frequency modulations in images of textured 3-D surfaces. A. A vertically corrugated surface overlaid with a horizontal-vertical plaid texture. B. The horizontal component of the plaid from A shown in isolation. C. The vertical component of the plaid from A shown in isolation. D. The same corrugated surface as in A overlaid with a complex plaid pattern consisting of eight iso-frequency gratings equally spaced in increments of 22.5 degrees.

Texture markings on a 3-D surface provide potentially useful cues to the 3-D shape of the surface when the surface is viewed in a 2-D image [1]–[11]. Local pattern changes across these 2-D images can be separated into modulations in orientation and spatial frequency, features for which it is well established that neurons early in the visual pathway are specialized. Examples are shown in Figure 1. In Figure 1a, a horizontal-vertical plaid texture is overlaid onto a surface that is corrugated in depth as a function of horizontal position. In the perspective image of the surface, oriented components of the texture that are parallel to the surface slant (in this example the horizontal grating component) converge and diverge forming patterns that we refer to as orientation flows (shown in isolation in Figure 1b), which exhibit minimal spatial frequency modulation. Although other oriented components also exhibit local changes in orientation, it is the pattern of flows formed specifically by the component running parallel to the surface slant that contains sufficient information to distinguish 3-D curvatures and that consistently conveys 3-D shape in isolation [12], [13]. The visibility of these orientation flows plays a critical role in correct shape perception [14]–[16]. Components perpendicular to the surface slant (here the vertical grating) are instead modulated in spatial frequency (Figure 1c) which in isolation can lead to misinterpretations about surface shape [14], [15], [17]. Given that most surface texture patterns contain multiple oriented components, the goal of the current study is to examine how the visibility of orientation flows for 3-D shape is affected by the presence of other oriented components in the surface texture.

Many surface textures contain components of roughly the same spatial frequencies at different orientations. When these iso-frequency patterns, such as the plaid in Figure 1a, are mapped onto a developable surface, any slant in the surface out of the fronto-parallel plane causes mismatches in frequency across components such that those that form the critical orientation flows maintain the lowest frequency across the image, and are thus more salient. Saliency increases with increasing surface slant as the frequency mismatch increases [16]. This difference in saliency is even more pronounced in iso-frequency patterns that have multiple frequency components such as the one shown in Figure 1d which contains eight gratings equally spaced in orientation. Thus, for iso-frequency texture patterns on developable surfaces, slanting a surface leads to an increase in visibility of the orientation flows and should facilitate the 3-D shape percept. It has been suggested that a form of frequency-selective cross-orientation suppression (COS) may contribute to this facilitation [16]. At the neural level, neurons responding to the local orientations along the orientation flows may be suppressed by the additional presence of components of the same frequency at other orientations [18]–[20]. The mismatch in the frequencies between the orientation flow components and these components created by slanting the surface may cause a *release* of frequency-specific COS. This could in turn lead to a decrease in neural suppression of the response to the orientation flows and thus an increase in their visibility and enhancement of perceived 3-D slant and shape.

**Figure 2:**
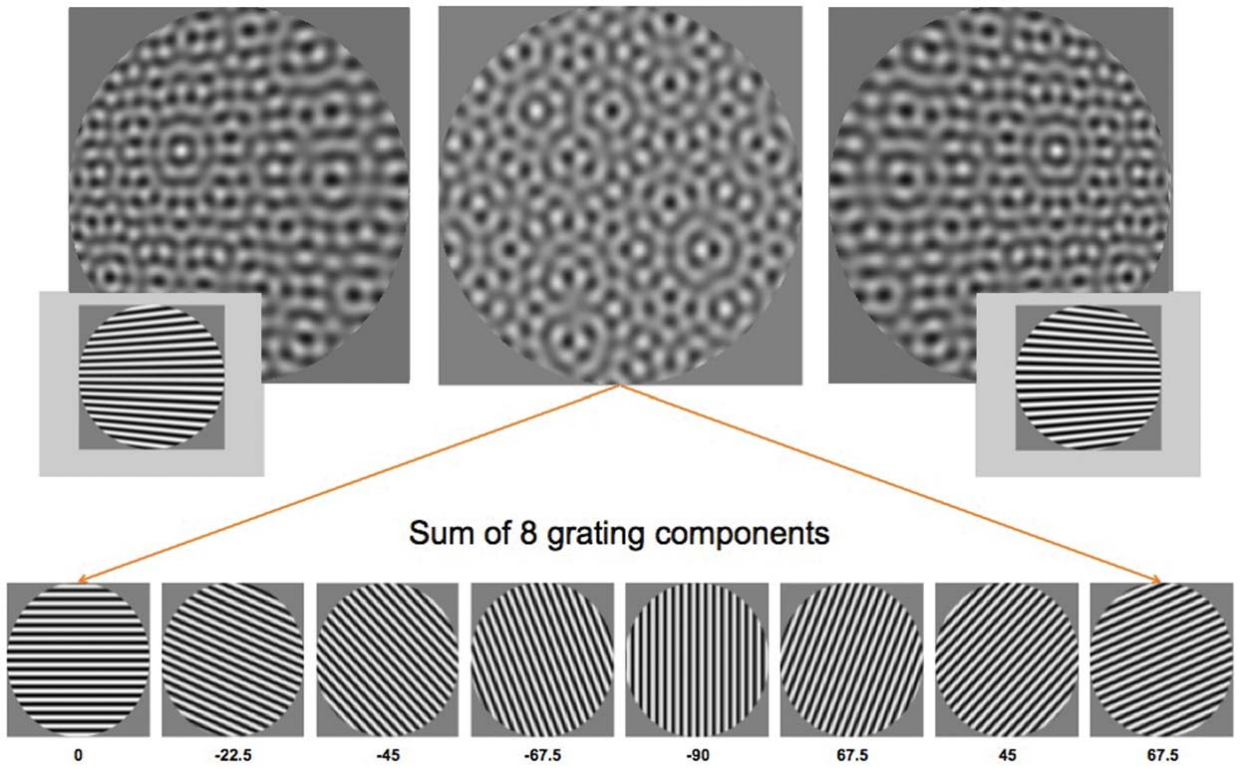
Complex plaid used in study consisting of 8 gratings equally spaced in orientation. When slanted out of the fronto-parallel plane, the component parallel to the surface slant creates the critical orientation flows. For left-right slanting surfaces, the horizontal component forms the orientation flows for the texture mapping used in this study. When all 8 components are equal in contrast, the flows are not visible. For demonstration purposes, the contrast of the flows has been enhanced in left and right images.

The current study aims to further investigate the factors that contribute to the visibility of orientation flows that are critical for 3-D shape perception by determining how the presence of components at different orientations affects orientation flow visibility in complex patterns. We are particularly interested in texture patterns and mappings for which slanting the surface does *not* result in increases in frequency of texture components, as is the case for developable surface mappings. If a COS mechanism contributes to 3-D shape perception as suggested by Li and Zaidi [16] and if it is physiologically and psychophysically broadband for orientation over a wide range of frequencies as found in previous work [18]–[23], then one would thus expect that texture components at any orientation, and at the same frequency as that which forms the orientation flows, to affect orientation flow visibility similarly. To test this prediction, we used the complex plaid pattern shown in Figure 2 consisting of eight iso-frequency gratings, systematically subtracted oriented components contained in the pattern, and measured contrast thresholds of orientation flows for planar slanted surfaces and curved concave and convex surfaces. Results consistently suggest that the visibility of orientation flows is selectively reduced by the presence of components that are closest to them in orientation. Thus unlike COS mechanisms previously found to be broadband in spatial frequency and orientation, any contribution of the COS mechanisms isolated here to the perception of 3-D shape from orientation flows appears to be both frequency- and orientation-specific.

## Materials and Methods

### 1. Apparatus and Presentation

All stimuli were presented on a calibrated 22 in. Mitsubishi Diamond Pro 2070 flat screen CRT monitor with a 1024×768 pixel screen at a refresh rate of 100 Hz. The monitor was driven by a Cambridge Research Systems ViSaGe Visual Stimulus Generator controlled via a 3.2 GHz Pentium 4 PC. Experimental code was written using the CRS Toolbox for Matlab. A CRS CB6 infrared response box was used to record responses.

Observers’ head positions were fixed with a chin-rest situated 1 m away from the stimulus monitor. All stimuli were presented at the center of the screen and the monitor was elevated such that the center of each image was level with the observer’s eye. Viewing was monocular; each observer patched the same eye across all sessions. An audio cue was the only feedback given to indicate that the observer’s response had been recorded. The experiment took place in a dimly lit room. To minimize fatigue, observers typically ran no more than two consecutive sessions and took breaks as they felt necessary. Sessions were randomized within and across observers.

### 2. Stimuli and Procedure

To examine whether proximity in orientation of neighboring components affects visibility of orientation flows, we generated a complex plaid texture and mapped it onto planar and corrugated surfaces. To isolate the effects of component orientation while minimizing the presence of frequency modulations in the image, we chose a different texture mapping from the developable surface mapping used in Figure 1. We used a volumetric carved solid texture mapping which creates identical orientation flows in the perspective image but which minimizes frequency modulations of other oriented components (see Li & Zaidi [15] for mapping details). The complex plaid textures were created by superimposing eight grating components at 2 cpd varying in orientation in 22.5 degree increments (0, ±22.5, ±45.0, ±67.5, and 90 degrees, where 0 deg is defined as horizontal). All components were subjected to the same texture mapping. When slanting the planar surface out of the fronto-parallel plane, only the component parallel to the surface slant creates the critical flows that contain sufficient information to distinguish 3-D curvatures [12], [13]. In this mapping, frequency modulations are minimized across components. For surfaces slanted around the vertical axis (left/right slants), the critical flows arise from horizontal components (Figure 2); whereas, the critical flows for surfaces slanted around the horizontal axis (floor/ceiling slants) arise from vertical components (this can be seen by rotating the images in Figure 2 by 90 deg). Visibility of the orientation flows was quantified by varying their contrast and determining the lowest contrast at which they were visible (the contrast threshold, CT). CTs were determined in the presence of the other components to evaluate their effect on orientation flow visibility. CTs of critical flows were measured for fronto-parallel surfaces and surfaces slanted ±60 degrees out of the fronto-parallel plane (examples shown in Figure 2). Additionally, similarly mapped sinusoidally corrugated surfaces were used to examine orientation flow visibility for more complex, curved surfaces. In the corrugated condition, each image contained 1.5 cycles of the sinusoidal depth corrugation, and the corrugations were simulated to span a depth amplitude of 14 cm.

**Figure 3:**
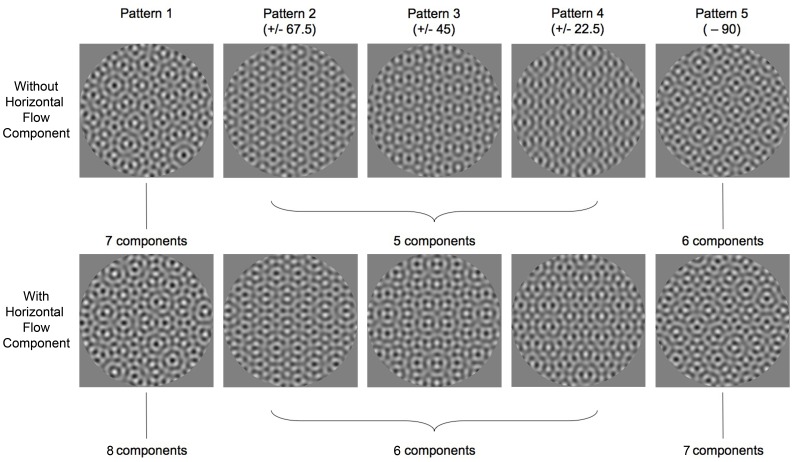
Five complex plaid patterns against which contrast thresholds of the orientation flows were measured. Patterns shown here were used in the left-right planar surface slant condition. Pattern 1 contains 7 gratings, each 22.5 deg from the next, excluding the horizontal (0 deg) grating. Pattern 2 is Pattern 1 less the 67.5 deg components. Pattern 3 is Pattern 1 less the 45 deg components. Pattern 4 is Pattern 1 less the 22.5 deg components. Pattern 5 is Pattern 1 less the 90 deg component.

CTs were measured in the presence of five different complex plaid textures shown in Figure 3. This figure shows patterns used for the left/right slanted condition (i.e. vertical slant axis). The top row shows patterns in the absence of the critical horizontal component and the bottom row shows the same patterns with the horizontal component added in. In the top row, Pattern 1 consists of seven oriented components (all but the horizontal that creates the orientation flows). In Patterns 2–5, we systematically subtracted components (indicated below each pattern label) from Pattern 1. For example, Pattern 4 was created by removing the components that are +22.5 and −22.5 degrees away from the critical flow component (here the horizontal component). Thus, it is the composite of ±45.0, ±67.5, and +90 degree components, and missing components closest in orientation to that of the flow component. Video frames containing the complex plaid patterns (Figure 3, top row) were interleaved with frames containing the critical flow component so that the contrast of this component could be independently manipulated. As a result of the interleaving, the contrast of each video frame could only be 50%. Thus the contrast of each grating component of the complex plaid frame was set to the maximum possible, 7.1%. For demonstration purposes, the critical horizontal flow component has been added at a contrast equal to that of all other components in the bottom panels of Figure 3.

All observers completed three experimental conditions: 1) planar surfaces slanted left/right about a vertical axis, 2) planar surfaces slanted along floor/ceiling slants about a horizontal axis, and 3) vertically corrugated surfaces varying in depth as a function of horizontal position. CTs of the flows were determined using a 3-down, 1-up staircase procedure, in which the contrast of the orientation flows was varied. In a single session, observers were presented with two randomly interleaved staircases (one ascending and one descending) for each of two complex plaid patterns ( = 4 staircases, 2 threshold estimates per pattern). Staircases were programmed to complete two reversals via 1.8% contrast steps followed by eight reversals via 0.4% contrast steps, from which contrast at the last six reversals were averaged as the estimate of threshold.

Each of the five patterns were blocked randomly into pairs for each session for each observer, and each pattern was tested in two separate sessions resulting in a total of 40 sessions across the three experimental conditions. Thus, each of the two planar conditions consisted of 15 sessions of randomly paired patterns at each of the three following slant conditions: 60 degrees positively slanted, 60 degrees negatively slanted, and fronto-parallel. The corrugated experimental condition contained 10 sessions of randomly paired patterns, in which there were five patterns for each of the two corrugations – convex and concave. As a result, four CT estimates for each pattern were averaged per observer.

Observers were seated 1 m away from the stimulus monitor and head position was fixed with a chinrest. Viewing was monocular in a dimly lit room. At the beginning of each session, a mean grey screen (58 cd/m^2^) was presented with a central fixation cross for 1 minute, which remained onscreen throughout the rest of the session. A tone signaled the start of the trials after the initial 1 minute adaptation. Stimuli were presented in circular apertures spanning 6.5 degrees against the mean grey background. In each trial of the experiment, observers were presented with two sequential stimuli for 500 ms each, separated by a 400 ms inter-stimulus interval, which were accompanied by audible tones of different frequencies. One of the stimuli was one of the complex plaid patterns without the orientation flows (e.g. panels in top row of Figure 3), and the other was the same complex plaid with the orientation flows (e.g. analogous panels in bottom row of Figure 3). The interval containing the flows was randomized across trials. Observers were asked to judge which of the two intervals contained the orientation flow pattern.

Observers were presented with written as well as verbal instructions regarding the experimental task, which included examples of visual stimuli and the patterns of orientation flows they were asked to detect, at very high contrast in the presence of various complex plaid patterns. Practice sessions were run for each of the three conditions for one of the pattern types (randomly chosen). Each session lasted approximately 15 minutes. Breaks were given between sessions to minimize visual fatigue.

### 3. Observers and Ethics Statement

The two authors and six naïve individuals (3 experienced but uninformed) served as observers in the experiment. All observers had normal or corrected-to-normal visual acuity. All research followed the tenets of the World Medical Association Declaration of Helsinki and informed written consent was obtained from the observers after explanation of the nature of the study. The research was approved by the Queens College Institutional Review Board.

## Results

We predict that if the orientation of neighboring components does not affect orientation flow visibility, CTs should be unaffected by the varying combinations of oriented components present in the pattern. If there is an effect of orientation proximity, then components that are closest in orientation to the critical flows should mask the flows more than components that are farther away in orientation. Therefore, subtracting those closest in orientation to the critical flow (Pattern 4) should cause a significant decrease in CTs.

An independent-measures ANOVA (F(4,315) = 28.83, *p*<.001) was conducted to compare CTs for each pattern collapsed across all surface conditions. Using the Bonferroni procedure for all pairwise comparisons, we found that CTs were significantly reduced when the components closest in orientation to that of the flows were subtracted out of the overall pattern (Pattern 4) reflecting the fact that the orientation flows were unmasked and thus most visible. This finding is inconsistent with the prediction that orientation flow visibility would be affected by components at all orientations equally. To further evaluate this finding, additional statistical tests were performed for each of the experimental conditions. These tests showed similar results. Per experimental condition, we used an independent-measures ANOVA to evaluate overall main effects. For each slant/corrugated surface, we used a repeated-measures ANOVA. All post-hoc pairwise comparisons were evaluated using the Bonferroni procedure. These are discussed below.

### 1. Planar Left/right Slanted Surfaces

**Figure 4:**
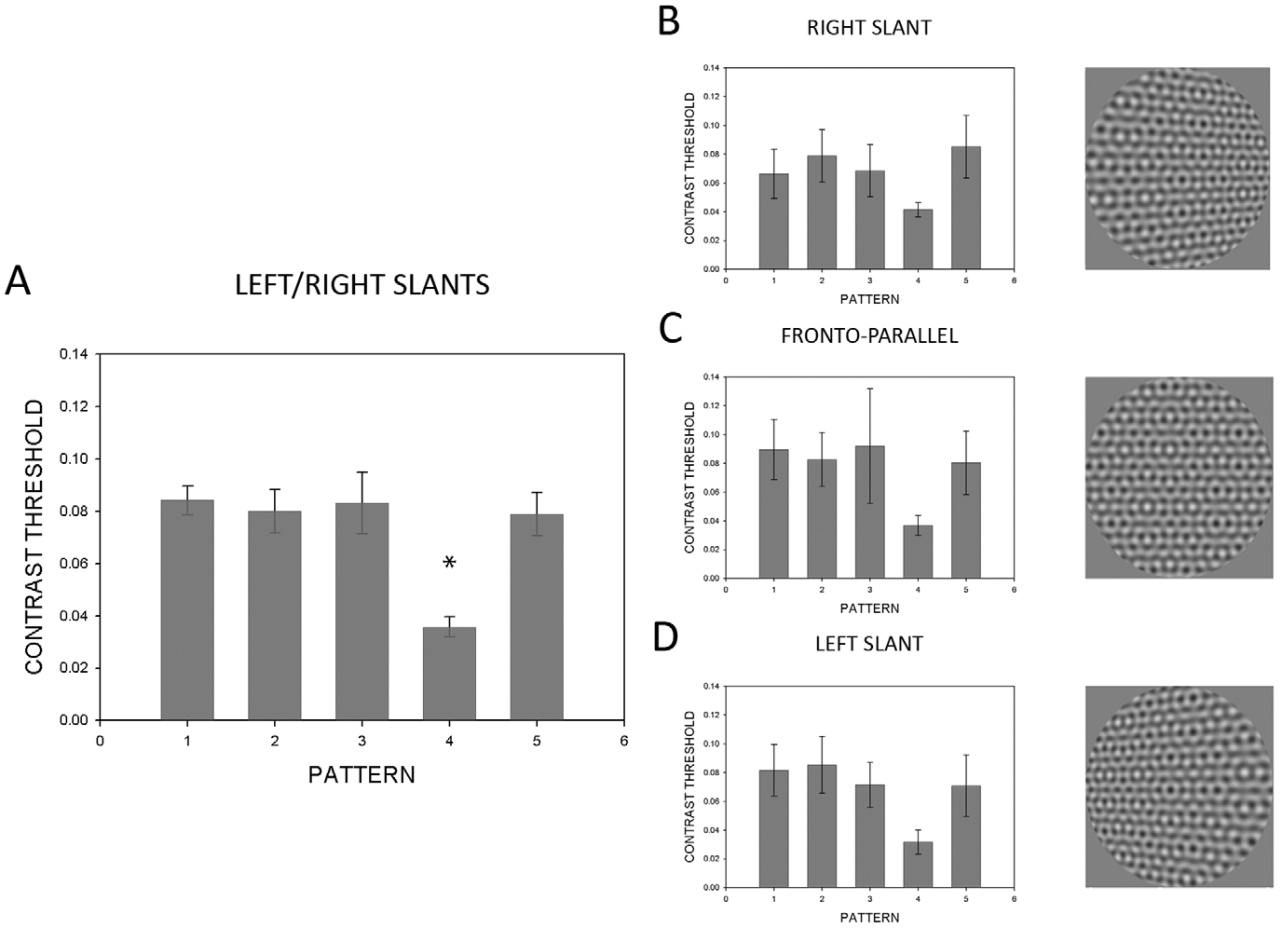
Mean contrast thresholds for the left-right planar slanted condition. The leftmost panel shows orientation flow CTs for each of the 5 patterns tested (see Figure 3) averaged across the fronto-parallel, +60 and −60 deg slanted conditions. Pattern 4 is the pattern missing the components closest in orientation to those of the orientation flows. CTs for each of the fronto-parallel, +60 and −60 deg conditions are shown on the right. Error bars represent 95% confidence intervals. Asterisks indicate significance at α<0.05.

Data for left/right slanted surfaces are presented in Figure 4. In the graph on the left (Figure 4A), CTs of the orientation flows were averaged across fronto-parallel and ±60 deg slanted surface conditions. An independent-measures ANOVA yielded a significant difference (*F*(4,115) = 11.14, *p*<.001) between pattern means. Post-hoc testing revealed significant differences only with respect to Pattern 4. For the graphs on the right (Figures 4B to 4D), subsequent analysis was conducted for each of the slant conditions separately – right slant, fronto-parallel, and left slant – using a repeated-measures ANOVA, which revealed significant differences (*F*(4,28) = 12.28, *p*<.001; *F*(4,28) = 15.02, *p*<.001; and *F*(4,28) = 5.21, *p* = .003, respectively). Post-hoc analysis found similar significant differences for Pattern 4, except between Patterns 3 and 4 when surfaces were slanted in either direction (Figure 4B and Figure 4D). Overall, these data indicate that the absence of neighboring components (or orientations closest to the orientation flows) allows for greater visibility of the orientation flows across surface slants.

### 2. Planar Floor/ceiling Slanted Surfaces

**Figure 5:**
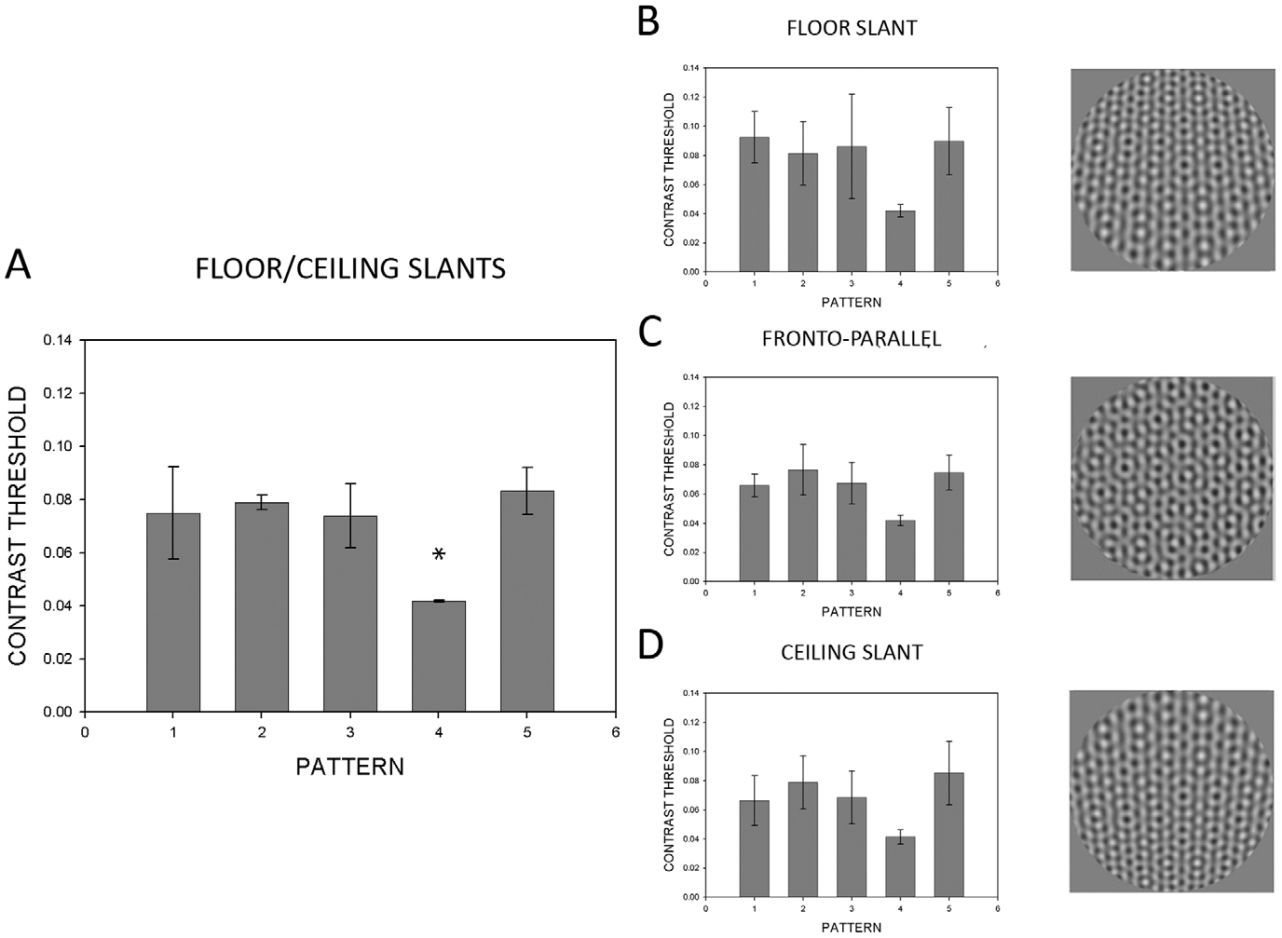
Mean contrast thresholds for the floor-ceiling planar slanted condition. See caption for Figure 4. Error bars represent 95% confidence intervals. Asterisks indicate significance at α<0.05.

To see if our results generalize to another slant axis, we ran an analogous condition for surfaces slanting around a vertical axis (Figure 5). An independent-measures ANOVA found significance (*F*(4,115) = 9.81, *p*<.001) for the overall main effects of the floor/ceiling slanted condition (left panel, Figure 5A). Using post-hoc analysis, significant differences were found only with respect to Pattern 4. Repeated-measures ANOVAs were performed per slant condition and resulted in the following significant findings: floor slant (*F*(4,28) = 7.99, *p*<.001), fronto-parallel (*F*(4,28) = 8.50, *p*<.001), and ceiling slant (*F*(2.71) = 12.31, *p*<.001). Post-hoc testing showed significant differences for Pattern 4, except between Patterns 3 and 4 for all surface slants (Figure 5A), Patterns 2 and 4 at fronto-parallel (Figure 5C), and Patterns 1 and 4 for the ceiling slanted surface condition (Figure 5D). Overall, the pattern of results for floor/ceiling slants is consistent with the pattern of findings for left/right slants.

### 3. Vertically Corrugated Surfaces

**Figure 6:**
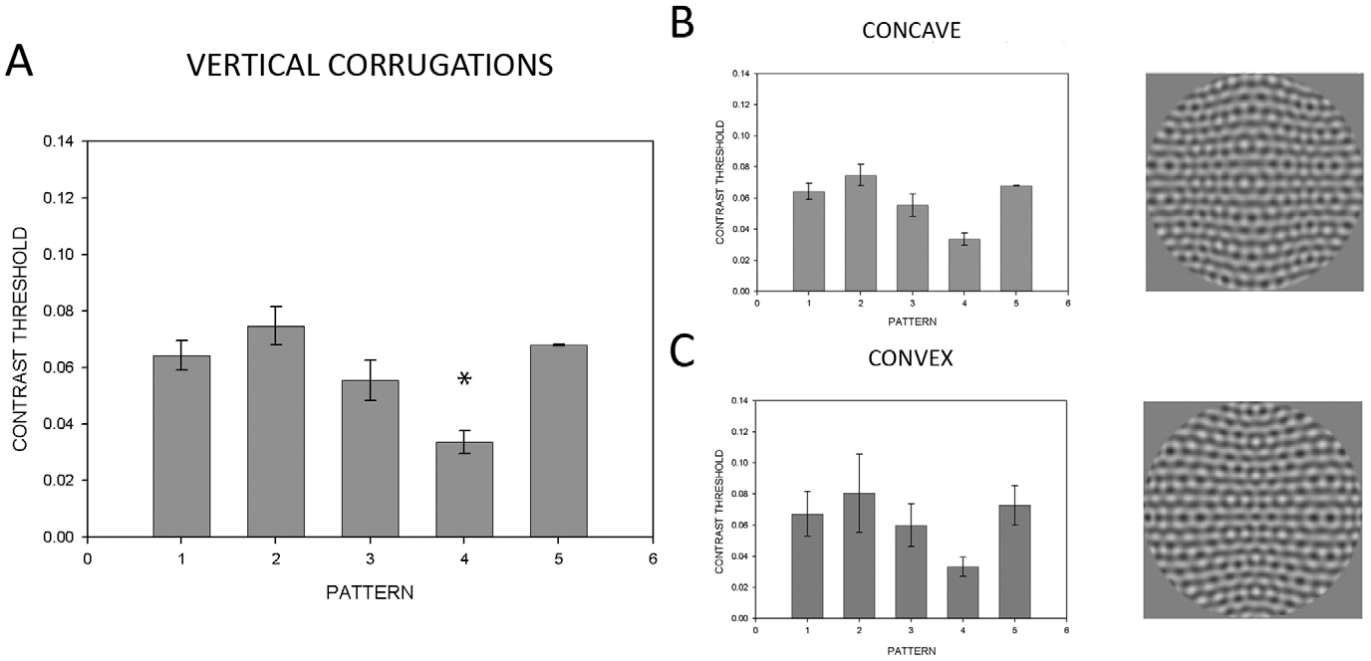
Mean contrast thresholds for the corrugated condition. The leftmost panel shows orientation flow CTs for each of the 5 patterns tested (see Figure 3) averaged across the concave and convex conditions. CTs for each of the concave and convex conditions are shown on the right. Error bars represent 95% confidence intervals. Asterisks indicate significance at α<0.05.

Finally, we also wanted to see if these findings generalized from planar surfaces to more complex, curved surfaces. We thus used vertically corrugated surfaces (varying sinusoidally in depth as a function of horizontal position) that were either centrally convex or concave. Vertically corrugated surfaces are composed of local left/right slants along the surface. Since these results should be consistent with those found for left/right slants, we predict the analogous pattern of results would be found between horizontally corrugated surfaces and our results for floor/ceiling slanted surfaces. In this final condition, the average of all flows produced a similar significant finding (*F*(4,115) = 9.89, *p*<.001) using an independent-measures design (Figure 6A). As previously found, post-hoc testing showed significant differences between patterns only with respect to Pattern 4. Moreover, individual curvature conditions (Figure 6, right panels) yielded significance for the pattern with the most proximal components subtracted out – convex (*F*(4,28) = 11.69, *p*<.001) and concave (*F*(4,28) = 9.00, *p*<.001), using a repeated-measures ANOVA. Subsequent analysis indicated significant differences only with respect to Pattern 4, except for Patterns 2 and 4 in the concave surface corrugation (Figure 6B).

To summarize across surface conditions, CTs are significantly reduced when components that are closest in orientation to that of the flows are subtracted from the pattern. This trend indicates that visibility of orientation flows that convey 3-D slant and curvature is most impaired by these proximal components. It is worth noting that for some surface conditions, CTs for Pattern 3 were not significantly different from the CTs for Pattern 4. Since ±45 deg components were subtracted from Pattern 3 and these components are the next most proximal in orientation to the orientation of the component creating orientation flows, it is not surprising that in some cases the removal of these components still has an effect on orientation flow visibility. However, when all surface conditions were averaged together (A panels in Figures 4, 5, 6) it is clear that CTs were consistently and significantly lowest for Pattern 4. It is also worth noting that the number of components across the five patterns tested is different (seven in Pattern 1, five in Patterns 2–4, six in Pattern 5). Despite the varying number of components, all patterns other than Pattern 4 contained the proximal ±22.5 deg components. This demonstrates not only the selective effect of the proximal components on orientation flow visibility, but the lack of effect on visibility of the number of components contained in the pattern, at least for the number of components tested in these patterns.

## Discussion

The goal of the current study was to examine the effects of orientation of non-critical texture components on the visibility of critical orientation flows for 3-D shape. We chose to examine this using complex plaid surface textures containing multiple spatial components, which might be more generally representative of naturalistic surfaces textures than simple plaids. Our results consistently show that, for planar and curved surfaces, orientation flow visibility is most affected by the presence of components oriented closest to them. Removal of these components caused significant increases in orientation flow visibility (as quantified by reductions in contrast threshold) while removal of all other less proximal components had no effect on their visibility.

For iso-frequency surface textures mapped onto developable surfaces, slanting the surface out of the fronto-parallel plane creates frequency mismatches between the texture components that create critical orientation flows and those that do not, thereby increasing the visibility of the orientation flows and increasing the strength of the slant percepts they convey. It has been suggested that the frequency mismatch increases orientation flow visibility via the release of a cross-orientation suppression (COS) mechanism that is frequency-selective [16]. Although COS has been previously implicated in visual processes such as orientation tuning [19], [24], [25], contrast gain control [20], [26]–[29], and the reduction of redundancy in the coding of natural images [30]–[32], this was the first suggestion of its potential contributions to 3-D shape perception.

The results of the current study, which utilizes a texture mapping that minimizes frequency modulations in texture components, compliment the results of Li and Zaidi [33] by showing that orientation flow visibility is not only affected by the frequencies of neighboring components, but also by their proximity in orientation such that components closer in orientation mask orientation flow visibility. Therefore, if a COS mechanism is contributing to our results, the process is not only frequency-selective, but also orientation-selective. However, given that many studies have found COS to be broadband in orientation [18]–[23], an additional or alternative mechanism that may be contributing to the visibility of orientation flows is surround suppression, which has been physiologically and psychophysically characterized as much being more narrowly tuned for orientation [20], [21], [34], [35]. The dimensions of our stimuli do not preclude the possibility that both types of suppression might be contributing to orientation flow visibility. The results also suggest that for complex texture patterns, the number of components present in the pattern has little effect on orientation flow visibility. Patterns 2–4 all contained five components, but only Pattern 4, for which the most proximal components were removed, showed significant decreases in contrast thresholds. Conversely, Patterns 1 and 5 respectively contained seven and six components (including the proximal components) and, as for Patterns 2 and 3 with fewer components, similarly had no effect on orientation flows visibility.

Although our study was not designed to parametrically quantify the orientation tuning of the underlying mechanisms at play, it is worth discussing our results in the context of orientation masking studies. In these studies, the tuning of perceptual orientation-selective mechanisms is quantified by measuring the visibility of a single test grating stimulus in the presence of a second overlaid masking grating stimulus of the same frequency but different orientation. Contrast thresholds of the test grating are typically greatest when the orientation of the mask matches that of the test, and falls off as this orientation difference increases. The tuning of the underlying mechanisms is characterized by the rate of this threshold fall-off. The tuning of the underlying mechanisms quantified in this way has been found to be on the order of 10–20 degrees, with several studies finding substantial decreases on test thresholds beyond about 12 degrees [36]–[38]. In light of these results, the fact that components 22.5 deg away from the orientation flows in our study had such a significant effect on their visibility may seem somewhat surprising. It is possible that the mechanisms isolated with masking studies are different from those contributing to orientation flow visibility in our study, which appear to be somewhat more broadly tuned. An important difference between the patterns used in this study and conventional masking studies is that ours contained multiple components. Thus, although the non-proximal components did not appear to have any effect on orientation flow visibility, perhaps orientation flow visibility in the presence of additional components requires the recruitment of additional mechanisms.

Another important difference between our stimuli and those used in masking studies is in the number of local orientations contained in the image. For our fronto-parallel conditions, all grating components were unmodulated in orientation and thus the difference in orientation between the orientation flows and the most proximal component was fixed at 22.5 deg across the image. For slanted and corrugated conditions, however, all components, including those that create orientation flows, contained multiple orientations across the image (e.g. see orientation flow patterns in inset panels of Figure 2). Thus there was in fact a range of orientation differences between the orientation flows and the most proximal components across the image. That said, our results for fronto-parallel, slanted, and corrugated conditions were qualitatively similar. Thus, it appears the effects of proximal components on orientation flow visibility are invariant to these orientation differences across the image.

Although not directly tested in this study, the importance of critical orientation flow patterns in the perception of 3-D shape has been substantiated in our previous work [12]–[15], [17], [39]–[41]. Together with Li and Zaidi [16], our results have important implications for image processing algorithms used to optimize 3-D shapes of surfaces and objects. Since orientation flow visibility is critical for 3-D shape perception, maximizing visibility is important. One way to maximize visibility is to ensure that oriented components of texture patterns be as different as possible in frequency from those that create orientation flows, or use a texture mapping (such as that used for developable surfaces) where surface slant creates frequency mismatches from those of the orientation flows. A second way to maximize visibility, as shown by the results of this study, is to ensure that the non-critical oriented components are at orientations that are far from those of the orientation flows. Surface textures with these spatial restrictions should convey the strongest 3-D slant and shape in projected images.
